# The Physiological Functions of the Golgin Vesicle Tethering Proteins

**DOI:** 10.3389/fcell.2019.00094

**Published:** 2019-06-18

**Authors:** Martin Lowe

**Affiliations:** Faculty of Biology, Medicine and Health, University of Manchester, Manchester, United Kingdom

**Keywords:** Golgi apparatus, golgin, vesicle traffic, tether, animal model, secretion, glycosylation, extracellular matrix

## Abstract

The golgins comprise a family of vesicle tethering proteins that act in a selective manner to tether transport vesicles at the Golgi apparatus. Tethering is followed by membrane fusion to complete the delivery of vesicle-bound cargo to the Golgi. Different golgins are localized to different regions of the Golgi, and their ability to selectively tether transport vesicles is important for the specificity of vesicle traffic in the secretory pathway. In recent years, our mechanistic understanding of golgin-mediated tethering has greatly improved. We are also beginning to appreciate how the loss of golgin function can impact upon physiological processes through the use of animal models and the study of human disease. These approaches have revealed that loss of a golgin causes tissue-restricted phenotypes, which can vary in severity and the cell types affected. In many cases, it is possible to attribute these phenotypes to a defect in vesicular traffic, although why certain tissues are sensitive to loss of a particular golgin is still, in most cases, unclear. Here, I will summarize recent progress in our understanding of golgins, focusing on the physiological roles of these proteins, as determined from animal models and the study of disease in humans. I will describe what these *in vivo* analyses have taught us, as well as highlight less understood aspects, and areas for future investigations.

## Introduction

**FIGURE 1:**
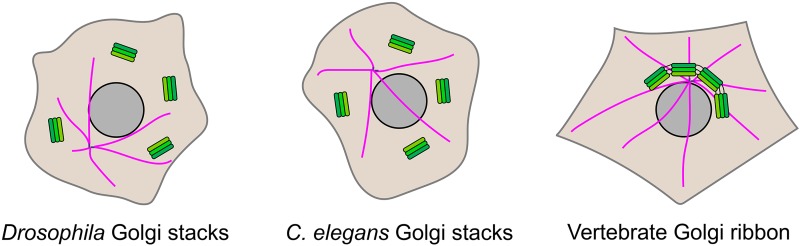
Golgi organization in different organisms. The Golgi apparatus is present as stacked cisternae in most organisms, including all metazoans. In the commonly used model organisms *Drosophila melanogaster* and *Caenorhabditis elegans* the Golgi stacks are present within the cytoplasm as discrete entities. In vertebrate species, the Golgi stacks are laterally connected by tubular continuities to generate a single-copy Golgi ribbon that is located adjacent to the centrosome. The Golgi stacks are shown in green, the centrosome in purple, and microtubules are in pink.

The Golgi apparatus lies at the heart of the secretory pathway, serving to modify newly synthesized cargo proteins and to sort and transport these proteins to their final destination, which may be inside or outside the cell. It is comprised of flattened membrane compartments called cisternae that are layered on top of one another to form the Golgi stack. In non-vertebrates the Golgi stacks exist as discrete entities within the cytoplasm, whereas in vertebrates the stacks are laterally connected to form a single-copy Golgi ribbon which is located adjacent to the centrosome (Figure 1). Newly synthesized cargo proteins arriving from the endoplasmic reticulum (ER) enter the Golgi apparatus at the *cis*-face, which in vertebrates comprises a tubulo-vesicular compartment called the *cis*-Golgi network (CGN). Cargo then transits the Golgi stack before arriving at the exit station of the Golgi, the *trans*-Golgi network (TGN), where it is sorted into carriers for delivery to its post-Golgi destination. As cargo transits the Golgi stack, numerous resident enzymes carry out post-translational modifications, most notably at the level of glycosylation, allowing for cargo maturation. There is extensive recycling of the resident enzymes between Golgi cisternae, which is required to maintain the distinct identity of the cisternae in the face of the cisternal migration that is thought to carry cargo forwards, and to ensure that cargo is correctly modified. In addition to the forward transport of newly synthesized cargo proteins, and the recycling of Golgi residents, the Golgi also receives recycling proteins from the endolysosomal system, which arrive at the TGN. We can therefore think of the Golgi apparatus as a hub for protein traffic. The golgins act in a selective manner to capture cargo protein-containing transport vesicles at different regions of the Golgi, in a process referred to as vesicle tethering. In this review, I will describe recent progress in understanding how golgins mediate vesicle tethering, and go on to describe how loss of golgin function can impact upon physiological processes, as determined from the use of animal models and the study of human diseases attributable to the loss of golgin function.

## Golgins as Vesicle Tethering Proteins

**FIGURE 2:**
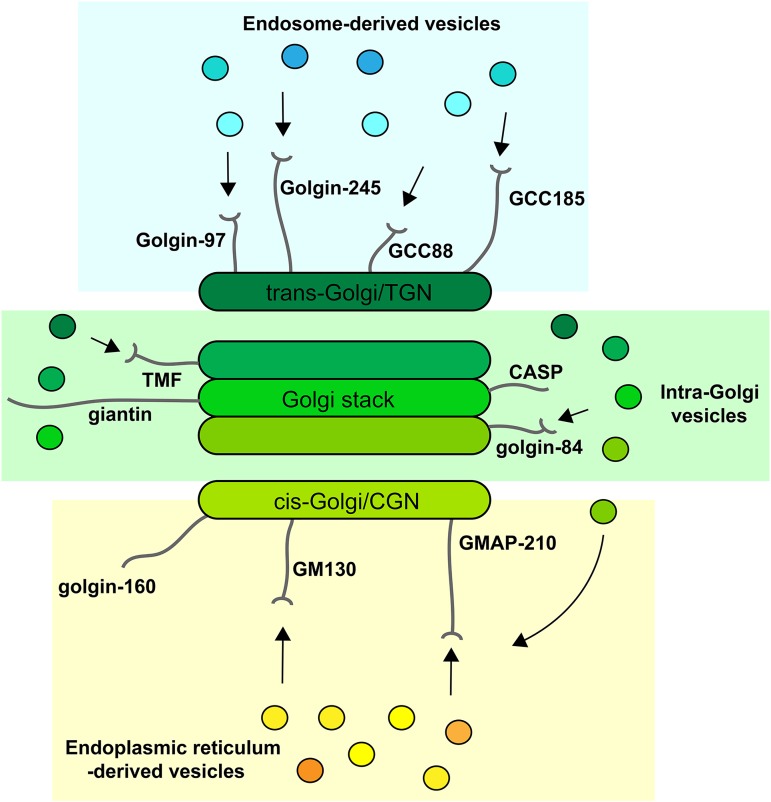
Golgins at the Golgi apparatus. Golgins are anchored to the Golgi membrane by their carboxy-terminus and protrude into the surrounding cytoplasm. They are localized to the *cis*-Golgi, which includes the *cis*-Golgi network (CGN) in vertebrates (yellow: GM130, GMAP-210, golgin-160), the rims of the Golgi stack (green: CASP, giantin, golgin-84, TMF), and the *trans*-Golgi/*trans*-Golgi network (TGN) (blue: GCC88, GCC185, golgin-97, golgin-245). For most golgins, the ability to tether vesicles has been demonstrated, indicated by arrows), but for some this ability remains to be shown (CASP, giantin, golgin-160). ER-derived vesicles are tethered at the *cis*-Golgi by GM130 and GMAP-210, which can also tether recycling intra-Golgi vesicles. Golgin-84 and TMF can only tether intra-Golgi vesicles, while the *trans*-Golgi golgins are able to ether endosome-derived vesicles.

The golgins comprise a family of Golgi-localized coiled-coil proteins with a similar topology (Munro, 2011; Witkos and Lowe, 2015). They are anchored to the Golgi membrane by their carboxy-terminus, and extend into the cytoplasm to facilitate vesicle capture, which is in most cases is mediated by the extreme amino-terminus of the protein (Cheung et al., 2015; Gillingham and Munro, 2016; Wong et al., 2017; Gillingham, 2018). In humans, there are at least 11 golgins, with varying degrees of conservation between different eukaryotes depending upon the particular golgin. The different golgins are localized to distinct regions of the Golgi apparatus, consistent with their ability to tether different vesicle types (Wong and Munro, 2014; Gillingham and Munro, 2016; Gillingham, 2018). For example, golgins localized at the *cis*-Golgi are competent to selectively tether vesicles arriving from the ER and intra-Golgi vesicles mediating recycling from later Golgi cisternae, whereas those at the *trans*-Golgi tether vesicles arriving from the endolysosomal system (Figure 2). In contrast, golgins residing within the Golgi stack are able to tether only intra-Golgi transport vesicles. Golgins therefore play a major role in dictating the specificity of vesicle traffic within the secretory pathway. In addition, the elongated nature of the golgins, coupled with their ability to tether vesicles via their membrane-distal amino-termini, giving a greater radius of capture, allows for increased efficiency of traffic. Following vesicle capture, golgins are thought to cooperate with other proteins, including Rab GTPases and multi-subunit tethering complexes such as COG and GARP, to mediate the transition from tethering to membrane fusion, which operates over a relatively short distance and is mediated by SNARE proteins (Witkos and Lowe, 2017).

Although the majority of mammalian golgins have been shown to mediate vesicle tethering, for a few members of the family this ability has yet to be formally demonstrated. This may reflect the nature of the cell-based assay that has been used to assess golgin-mediated tethering, which may have missed tethering interactions mediated by certain golgins (Wong and Munro, 2014; Wong et al., 2017), or it could indicate that some golgins have functions other than vesicle tethering. Golgin-160, for example, has been shown to mediate binding to the microtubule motor protein dynein for Golgi morphology and positioning (Yadav et al., 2012). This does not preclude an additional vesicle tethering activity for golgin-160, but such an activity has so far not been demonstrated. Other golgins have also been shown to recruit factors to the Golgi membrane in order to control downstream cellular processes. GM130 is particularly interesting in this regard. In addition to acting as a tether for ER-derived vesicles (Wong and Munro, 2014), it also interacts with the microtubule nucleation factor AKAP450 to promote microtubule nucleation at Golgi membranes (Rivero et al., 2009), the protein kinase STK25, which is important for cell migration (Preisinger et al., 2004), RasGRF, a Cdc42 GEF also important for cell migration (Baschieri et al., 2014), and WAC, which functions in autophagy induction (Joachim et al., 2015). It is therefore possible for golgins to have cellular functions other than, or in addition to, vesicle tethering, although for most golgins it would appear that vesicle tethering is their primary role.

## Animal Models for Golgin Function

As might be expected, numerous cell-based studies have been carried out to investigate the roles of golgins in vesicle trafficking. Loss of function studies have typically been performed in generic cell lines, using model cargo proteins, to give us a picture of what trafficking steps different golgins participate in (Ramirez and Lowe, 2009; Munro, 2011; Cheung and Pfeffer, 2016). The use of generic cell lines and model cargoes for these studies is certainly justified considering that the golgins are widely expressed throughout tissues. However, in many cases it has not been possible to unambiguously determine the functional requirement for a golgin using this type of approach, with relatively mild phenotypes reported, or in some cases no trafficking phenotypes at all. A possible explanation is functional redundancy between the golgins. For example, the *trans*-Golgi golgins share overlap in their ability to tether vesicles containing the same endosomal cargo proteins, while GMAP-210, golgin-84, and TMF can tether vesicles with the same intra-Golgi cargo (Wong and Munro, 2014; Wong et al., 2017). This is consistent with these golgins sharing similar vesicle tethering motifs in their amino-termini, which are distinct from those found in the *trans*-Golgi golgins (Wong and Munro, 2014; Wong et al., 2017). Hence, loss of a single golgin may not manifest as a phenotype due to redundancy with another golgin. The use of model cargo proteins may also fail to reveal the requirement for a golgin in the trafficking of a particular cargo, which may have a greater dependency on one golgin compared to others. Moreover, the organization of the secretory pathway can vary between cell types, which again may allow for differential requirements for golgins in trafficking within cells. Finally, golgins may only enhance trafficking efficiency, such that loss of a golgin may only have a minimal impact upon rates of transport as measured in the standard trafficking assays used in cultured cell models. Hence, the inherent limitations of model cell lines and cargo proteins make it difficult to appreciate the functional requirement for golgins in trafficking in a broader context, and do not allow for assessment of the physiological consequences of impaired golgin-dependent transport. One way to address these issues is to use animal models, allowing the analysis of the myriad different cell types throughout the body, and the cargo proteins expressed by these cells, as well as determining the consequences upon organismal physiology upon loss of golgin function. Such approaches can also reveal degrees of functional redundancy in an *in vivo* context. The established animal models for golgins, and the information that has been gained from them, are discussed below (see also Table 1).

**Table 1:** Animal models and human diseases associated with loss of golgin function.

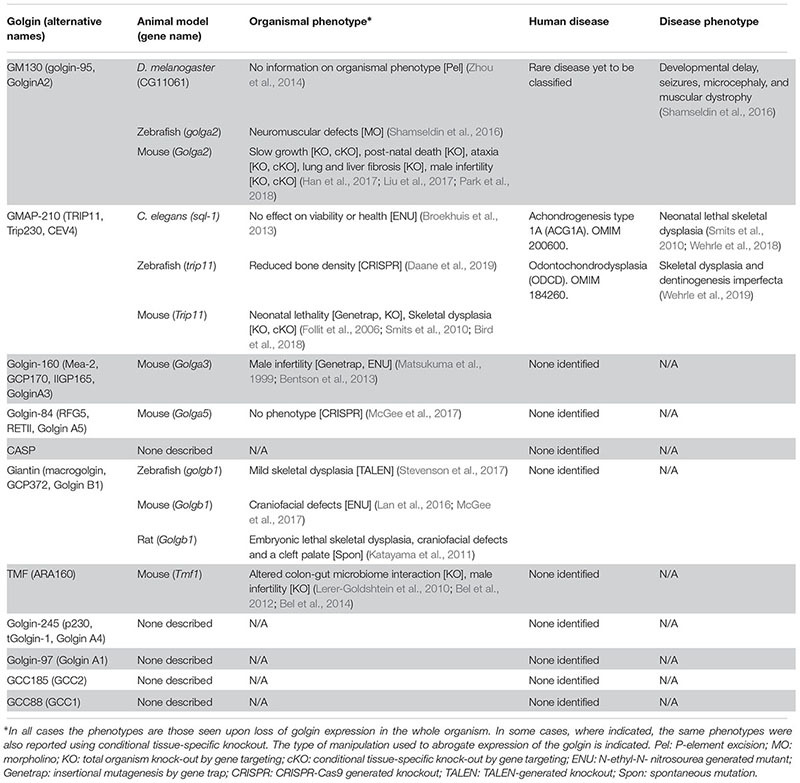

### Golgins at the *cis*-Golgi

#### GM130

One of the best-studied golgins is GM130, which is anchored to the *cis*-Golgi via binding to the Golgi stacking protein GRASP65 (Nakamura et al., 1995; Barr et al., 1997), although interactions with the related GRASP55 and Rab1 may also contribute to its Golgi targeting (Moyer et al., 2001; Short et al., 2001; Weide et al., 2001; Bekier et al., 2017). GM130 is able to tether ER to Golgi vesicles at the *cis*-Golgi (Wong and Munro, 2014), and, as described above, can anchor a number of other proteins, namely AKAP450, STK25, RasGRF, and WAC, to the Golgi membrane (Preisinger et al., 2004; Rivero et al., 2009; Baschieri et al., 2014; Joachim et al., 2015). Depletion of GM130 in cultured cells has given variable results, with some studies reporting no effects upon transport (Puthenveedu and Linstedt, 2001; Kondylis and Rabouille, 2003; Puthenveedu et al., 2006), whereas others have seen reduced rates of ER to Golgi transport (Marra et al., 2007; Diao et al., 2008), although in most cases a loss of Golgi ribbon architecture is observed. GM130 has been knocked out in the fruit fly *Drosophila melanogaster* (Zhou et al., 2014). The flies appear viable, although a detailed phenotypic analysis has yet to be reported. As seen in mammalian hippocampal neurons (Horton et al., 2005), the Golgi can exist as “outposts” within the dendritic arbor of *Drosophila* larval neurons (Zhou et al., 2014). Loss of GM130 within the larval neurons causes loss of Golgi outpost organization, and a reduced ability to nucleate microtubules from the outposts, accompanied by reduced dendritic branching, indicating a role for the protein in neuronal Golgi and microtubule organization.

**FIGURE 3:**
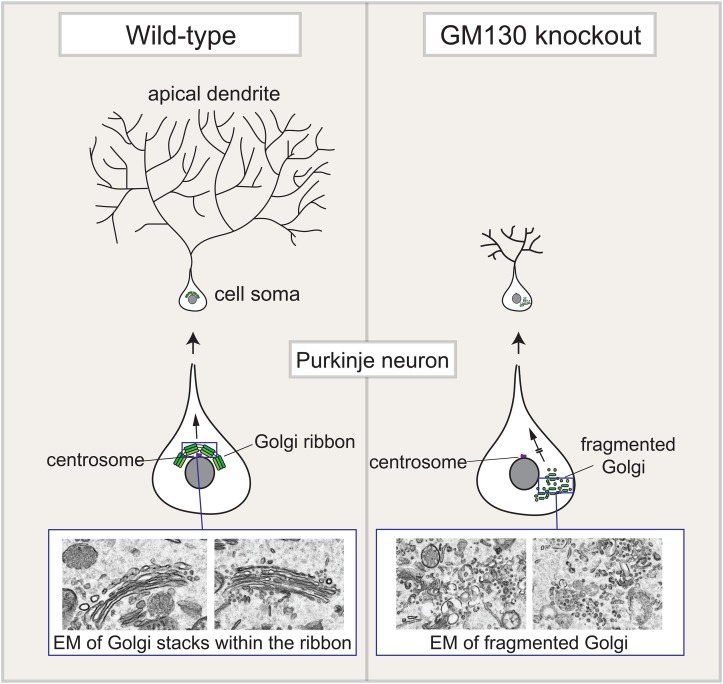
Golgi fragmentation in Purkinje neurons upon loss of GM130. The Golgi apparatus in Purkinje neurons is located within the cell soma, close to the centrosome, at the base of the elaborate apical dendritic tree that forms in these neurons. This positioning allows for polarized delivery of newly synthesized secretory cargo to the dendrite, which is required for its growth and maintenance. Loss of GM130, either in the whole organism, or selectively with the central nervous system, causes fragmentation of the Purkinje Golgi apparatus and its mislocalization away from the centrosome. Golgi fragmentation is shown both schematically and in electron microscope (EM) images of the Purkinje neuron Golgi apparatus. These changes result in reduced cargo delivery into the dendrite, which causes atrophy of the dendritic tree. This ultimately causes death of the Purknije neurons, which control movement, and as a consequence the GM130 knockout mice suffer from severe ataxia.

Morpholino-induced depletion of GM130 in zebrafish larvae causes reduced brain size, skeletal muscle disorganization, and altered mobility, suggesting a neural or neuromuscular defect (Shamseldin et al., 2016), although the mechanistic basis for these phenotypes remains to be determined. Loss of mammalian GM130 also leads to a major neurological phenotype (Liu et al., 2017). Although viable at birth, GM130 knockout mice fail to thrive and die by 1–2 months of age. Loss of GM130 globally, or by tissue-specific knockout within the central nervous system, results in progressive neurodegeneration due to death of Purkinje neurons within the cerebellum, which causes ataxia. Purkinje neurons have a large and extensively branched primary dendrite, which requires constant delivery of protein and lipid for its growth and maintenance, which continues into post-natal development (Dvorak and Bucek, 1970; Tanaka, 2009). The Golgi is particularly well developed in Purkinje neurons and is positioned at the base of the dendrite within these cells, allowing for extensive and polarized delivery of secretory cargoes into the dendrite (Tanabe et al., 2010; Liu et al., 2017). Upon loss of GM130, the Purkinje cell Golgi is mis-localized, possibly through loss of binding to AKAP450, and the Golgi ribbon is fragmented (Liu et al., 2017) (Figure 3). These changes are accompanied by reduced secretory traffic into the dendrite, accounting for the atrophy of the dendritic arbor seen in these cells. Whether impairment of microtubule nucleation at the Golgi contributes to the Purkinje cell phenotype is currently unclear, as is any potential contribution of Golgi outposts, which appear to be absent from this type of neuron. Nevertheless, the mouse studies point to an important role for GM130 in neuronal Golgi organization and trafficking, which is important for the maintenance of Purkinje cells. Whether other neuronal types in mammals are also affected by loss of GM130, as suggested by the study of *Drosophila* and zebrafish GM130, remains to be seen.

Loss of mouse GM130 affects Golgi morphology in other tissues, with fragmentation of the Golgi ribbon seen in two major secretory cell types, the pancreatic acinar cell and the type II alveolar cell within the lung (Liu et al., 2017). Further work will be required to determine the physiological consequences of GM130 loss in these cells. The study of a second GM130 knockout mouse model, which also has reduced viability and growth, has revealed changes in liver and lung pathology, with increased numbers of autophagosomes and increased fibrosis within these tissues (Park et al., 2018). How the loss of GM130 results in these phenotypes remains to be determined. Finally, loss of GM130 in mice, both globally or using a germ-cell tissue specific knockout, causes defects in spermatogenesis, resulting in male infertility (Han et al., 2017). During spermatogenesis, the Golgi plays an important role in biogenesis of the acrosome, which is a lysosome-related organelle located at the head of the sperm (Moreno et al., 2000). The acrosome contains digestive enzymes that, upon release, break down the outer layers of the ovum to allow fertilization to occur. Hence defective acrosome biogenesis often results in male infertility. GM130 knockout mice are completely deficient in acrosome biogenesis, explaining the penetrant male infertility phenotype seen in these animals (Han et al., 2017). Interestingly, acrosome biogenesis is particularly sensitive to perturbation of Golgi function, and this process is defective in animals lacking several other golgins (see below).

#### GMAP-210

GMAP-210 is bound to the *cis*-Golgi membrane through interaction of its carboxy-terminal GRAB domain with the small GTPase Arf1 (Gillingham et al., 2004). It is competent to tether both ER-derived transport vesicles and retrograde intra-Golgi transport vesicles via two overlapping vesicle-tethering motifs in its amino-terminus (Drin et al., 2008; Wong and Munro, 2014; Wong et al., 2017). It can also anchor the ciliary protein IFT20, which is a component of the IFT (intraflagellar transport) complex, to the Golgi (Follit et al., 2006). Through this interaction it is likely that GMAP-210 participates in the trafficking of cargo proteins from the Golgi to the cilium (Monis et al., 2017), although it has also been proposed that IFT20 participates in ER to Golgi transport (Noda et al., 2016). In both cases the mechanistic details remain to be dissected. Loss of GMAP-210 at the cellular level results in extensive Golgi fragmentation and reduced rates of secretory traffic (Smits et al., 2010; Roboti et al., 2015; Sato et al., 2015; Wehrle et al., 2018). Several animal models for GMAP-210 have been generated, and the phenotypes are consistent with defects in Golgi and/or cilia function. Loss of the GMAP-210 ortholog in *Caenorhabditis elegans*, called SQL-1, does not affect health or viability, but there is mild disruption of Golgi organization, and defective IFT transport, most likely as a consequence of reduced delivery of components required for IFT transport from the Golgi to the cilium (Broekhuis et al., 2013).

Both global and tissue-specific knockout mouse models have been generated to study mammalian GMAP-210, also called TRIP11. Systemic loss of GMAP-210 results in neonatal lethality, indicating that GMAP-210 is an essential protein (Follit et al., 2006; Smits et al., 2010). In the first knockout model, which utilized a gene trap to disrupt GMAP-210 expression in all tissues, there is a pleiotropic phenotype with defects in the heart and lung, as well as reduced overall growth (Follit et al., 2006). Although the underlying cause of these phenotypes remains to be determined, it has been proposed that impaired cilium-based signaling during development may be responsible. The most prominent phenotype in the second knockout model, which was an *N*-ethyl-*N*- nitrosourea (ENU)-generated mutant, was skeletal dysplasia, with shortening of the bones and reduced ossification (Smits et al., 2010). These changes were accompanied by abnormal chondrocyte differentiation and increased cell death. The chondrocyte Golgi apparatus is extensively fragmented upon loss of GMAP-210, and there is a deficit in glycosylation and secretion of extracellular matrix proteins. The reduced ability to modify and secrete matrix proteins is likely to account for the bulk of the cartilage and bone phenotype seen upon in the GMAP-210 knockout mice. However, it also possible that altered ciliary signaling could contribute to the phenotypes considering that signaling from the cilium is also important for various aspects of bone morphogenesis, including chondrocyte differentiation and formation of the growth plate (Braun and Hildebrandt, 2017). Interestingly, the Golgi was fragmented in other embryonic tissues e.g., kidney, but not in others e.g., lung and intestine, indicating differences in the requirement for GMAP-210 to maintain Golgi organization between cell types (Smits et al., 2010). More recently, GMAP-210 has been conditionally knocked out in various cell types associated with skeletal development, which showed that it is the loss of GMAP-210 in chondrocytes, as opposed to fibroblasts, osteoclasts or osteoblasts, that is important for the skeletal phenotype seen in GMAP-210-deficient mice (Bird et al., 2018). This study also showed that secretion of certain matrix proteins was more sensitive to loss of GMAP-210 that others, supporting the view that GMAP-210 may promote the trafficking of certain cargoes over others. More recently, it has been shown that loss of Gmap-210 from zebrafish gives a skeletal phenotype, with a reduction in bone density (Daane et al., 2019). This suggests the physiological role of GMAP-210 is conserved between vertebrates, although further characterization will be required to confirm this.

#### Golgin-160

Golgin-160 (also known as GOLGA3) is enriched towards the *cis*-side of the Golgi apparatus (Hicks et al., 2006). It has been shown to recruit the dynein microtubule motor protein to Golgi, which is important for Golgi positioning and ribbon maintenance (Yadav et al., 2012), but whether it participates in vesicle tethering has yet to be demonstrated. Loss of golgin-160 in two different mouse models has no effect upon viability (Matsukuma et al., 1999; Bentson et al., 2013), which is perhaps surprising considering the strong Golgi positioning defect seen when golgin-160 is depleted in cultured cells (Yadav et al., 2012). The only overt phenotype *in vivo* is male sterility (Matsukuma et al., 1999; Bentson et al., 2013). There is increased death of male germ cells upon loss of golgin-160, and those sperm that are generated have deficient acrosome biogenesis (Bentson et al., 2013). Further analysis is required to ascertain the cause of death attributable to loss of golgin-160, which has previously been implicated in pro-apoptotic signaling, and to determine how its loss impairs acrosome biogenesis, although it is likely via defective Golgi positioning or trafficking.

### Golgins at the Cisternal Rims

#### Golgin-84 and CASP

Golgin-84 is present at the cisternal rims of the Golgi apparatus, where it is enriched towards the *cis*-side (Diao et al., 2003; Satoh et al., 2003). Golgin-84 is able to tether intra-Golgi transport vesicles, and as a transmembrane protein, is also recycled to earlier cisternae in intra-Golgi transport vesicles, in which case it is a cargo protein (Wong and Munro, 2014). There are orthologs of golgin-84 in *D. melanogaster* and *C. elegans*, although they have yet to be analyzed at the functional level. Mice lacking golgin-84, also known as GOLGA5, are viable, and there is no overt phenotype in these mice, which develop and grow normally (McGee et al., 2017). There is also no male infertility, indicating that golgin-84 is dispensable for acrosome biogenesis. The golgin CASP is also anchored by a carboxy-terminal transmembrane domain and present at the cisternal rims (Gillingham et al., 2002). CASP can bind golgin-84, and although this interaction has been implicated in vesicle tethering (Malsam et al., 2005), it remains to be demonstrated whether CASP is sufficient to tether vesicles in its own right. Interestingly, and in contrast to golgin-84, CASP is absent from *D. melanogaster*. No animal knockout for CASP has been generated as of yet so the *in vivo* importance of CASP remains to be determined.

#### Giantin

Another golgin present at the cisternal rims is giantin, the largest golgin, which, like golgin-84 and CASP, is anchored to the Golgi membrane by a carboxy-terminal transmembrane anchor (Linstedt et al., 1995). It has yet to be shown that giantin can tether transport vesicles in cells. If it does, then it is likely to tether certain types of intra-Golgi vesicles (Sonnichsen et al., 1998), although it may also play a role in laterally linking Golgi cisternae within the Golgi ribbon (Koreishi et al., 2013). Loss of giantin in cultured cell models gives a mild cilia and secretory trafficking phenotype, with little apparent effect on Golgi morphology (Koreishi et al., 2013; Bergen et al., 2017). Giantin is absent from *D. melanogaster* and *C. elegans*, but several vertebrate models have been generated to study the functions of the protein *in vivo*. Loss of giantin in zebrafish manifests as changes in cilia number and morphology (Bergen et al., 2017). Although the underlying mechanism remains to be determined, it is possible defective trafficking to the cilium may be responsible for this phenotype. There is also a mild skeletal phenotype in giantin-deficient zebrafish, which is reminiscent of a congenital disorder of glycosylation known as hyperphosphatemic familial tumoral calcinosis (HFTC) in humans (Stevenson et al., 2017). This phenotype may be explained by the loss of expression of the glycosyltransferase GALNT3 in the giantin-deficient zebrafish, whose mutation in humans causes HFTC (Topaz et al., 2004). Interestingly, GALNT3 is one of 22 glycosyltransferases whose expression is altered upon loss of giantin, which is indicative of an adaptive or compensatory response to chronic loss of the protein. This result lends support to the idea that giantin is important for Golgi function, possibly in enzyme recycling within the Golgi stack, and also indicates that chronic loss of a Golgi protein can induce adaptive or compensatory responses to alleviate the phenotype. It also raises the possibility that adaptive or compensatory responses can account for lack of phenotypes seen in other golgin knockout models, either within certain tissues and/or throughout the organism.

Loss of mammalian giantin, also called GOLGB1, in mouse knockout models, has revealed craniofacial defects, including a cleft palate (Lan et al., 2016; McGee et al., 2017). There is reduced accumulation of the matrix glycosaminoglycan (GAG) hyaluronan and reduced protein glycosylation, consistent with giantin functioning to maintain cargo protein glycosylation and GAG synthesis at the Golgi, which in turn is important for proper matrix assembly and formation of the palate (Lan et al., 2016). Thus, although giantin is widely expressed throughout the body, the knockout phenotype is restricted to certain tissues. The combined knockout of giantin and golgin-84 gives the same phenotype as loss of giantin alone, arguing against the possibility that these golgins function in a redundant manner *in vivo* (McGee et al., 2017). In contrast to knockout mouse models, rats lacking giantin display a much more severe phenotype, manifesting as an embryonic lethal osteochondrodysplasia, with systemic oedema and shortening of the long bones in addition to craniofacial defects and a cleft palate (Katayama et al., 2011). The chondrocytes from these animals have a swollen ER and disrupted Golgi, reduced GAG production, and altered production of extracellular matrix proteins (Kikukawa et al., 1990, 1991; Katayama et al., 2018). Thus, giantin would appear to be important for proper glycosylation and possibly secretion of extracellular matrix proteins and GAGs, which in turn is important for skeletal development, albeit with varying degrees of phenotypic severity in the different vertebrate models.

#### TMF

TMF is also present at the rims of the Golgi cisternae, but is more enriched towards the *trans*-side of the Golgi compared to golgin-84 and giantin, consistent with its recruitment to the membrane by Rab6 (Fridmann-Sirkis et al., 2004; Yamane et al., 2007). TMF is competent to tether intra-Golgi transport vesicles (Wong and Munro, 2014), which is mediated by an amino-terminal tethering motif, and unlike other golgins, there is also a separate tethering motif lying within the central region of the protein (Wong et al., 2017). Depletion of TMF from cells results in altered Golgi morphology and displacement of the Golgi enzyme GalNac-T2, consistent with a role in retrograde intra-Golgi transport (Fridmann-Sirkis et al., 2004; Yamane et al., 2007). There are orthologs of TMF in *D. melanogaster* and *C. elegans*, although they have yet to be analyzed at the functional level. TMF knockout mice are viable and healthy, although male mice are sterile (Lerer-Goldshtein et al., 2010). Loss of TMF does, however, affect the composition of mucus within the colon, which is comprised of the heavily glycosylated mucin proteins. There is altered post-translation modification and secretion of the MUC2 mucin from goblet cells, which in turn affects interaction between the colonic epithelium and gut microbiome (Bel et al., 2012, 2014). The mechanism by which loss of TMF alters MUC2 processing and trafficking awaits further investigation, but is likely to involve disrupted recycling of Golgi enzymes within the Golgi stack. The male sterility phenotype in TMF knockout mice arises from defective acrosome biogenesis. The Golgi is mis-positioned in developing spermatids upon loss of TMF, and there is reduced tethering of pro-acrosomal vesicles, explaining the lack of a functional acrosome in these cells (Lerer-Goldshtein et al., 2010; Elkis et al., 2015). The results are consistent with a role for TMF in membrane delivery into the forming newly forming acrosome during spermatogenesis.

### Golgins at the *Trans*-Golgi

There are four golgins at the *trans*-Golgi that function in vesicle tethering at this compartment, namely golgin-245, golgin-97, GCC185 and GCC88 (Cheung and Pfeffer, 2016; Gillingham and Munro, 2016). They are all anchored to the *trans*-Golgi membrane via a carboxy-terminal GRIP domain, which binds to membrane-associated Arl1 (Munro, 2011; Witkos and Lowe, 2015). GCC185, in addition to a vesicle trafficking function, also contributes to microtubule nucleation at the Golgi through binding to the microtubule binding proteins CLASP1 and CLASP2 (Efimov et al., 2007). There appears to be overlapping functionality in vesicle trafficking between the GRIP domain golgins in that they can tether vesicles carrying the same endosome-derived model cargo protein CI-MPR (Wong and Munro, 2014; Cheung et al., 2015). However, it should be noted that the same cargo protein may be carried by more than one type of transport vesicle. Indeed, it was recently demonstrated that GCC88 tethers only a sub-population of vesicles carrying CI-MPR, which are presumably different to the CI-MPR containing vesicles tethered by the other *trans*-Golgi golgins (Cui et al., 2019). This finding is consistent with the different vesicle-tethering motif found in GCC88 compared to that in the other golgins. In this regard, golgin-245 and golgin-97 share a common tethering motif, binding to the RabGAP-like protein TBC1D23, which in turn binds the WASH complex on endosome-derived vesicles to mediate tethering (Shin et al., 2017). Although all of the GRIP-domain golgins have an ortholog in *D. melanogaster*, and all except golgin-97 have an ortholog in *C. elegans*, the functional requirement for these golgins in either model organism has yet to be tested. As of yet, no vertebrate knockout models for any of the GRIP-domain golgins have been generated.

## Loss of Golgin Function as a Cause of Human Disease

With the advent of “next-generation sequencing” (NGS), there has been an explosion in the discovery of rare genetic variants responsible for human disease (Boycott et al., 2013). Amongst the many pathogenic variants that have been discovered are those within genes encoding Golgi proteins (Zappa et al., 2018). The identification of these rare variants, and the study of patients carrying them, are important not only from a clinical perspective, but can also prove extremely informative with regard to deciphering the physiological roles of the encoded proteins. We may expect the phenotypes to mirror those seen in animal models, but this is not always the case, which can be due to a variety of reasons, including differences in organismal physiology, the expression of compensatory genes, or adaptive responses in the different organisms. To date, mutations in only two of the golgins, GMAP-210 and GM130, have been linked to disease in humans, as described further below(see also Table 1).

### GMAP-210

**FIGURE 4:**
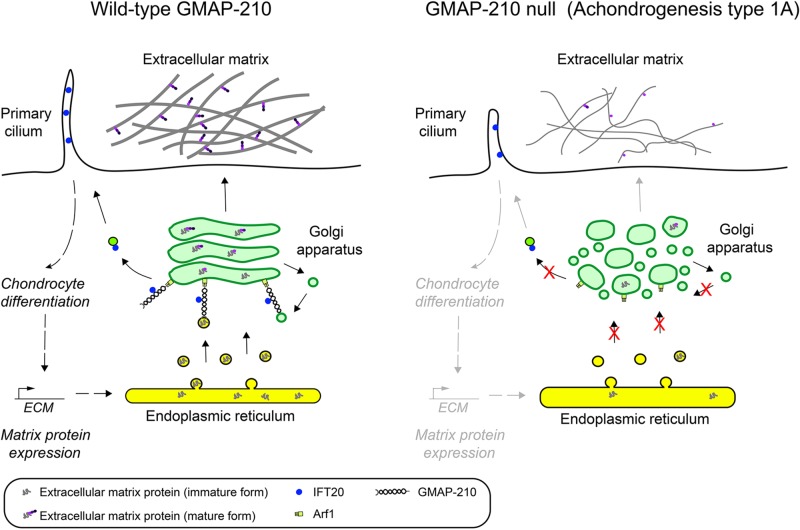
Loss of GMAP-210 manifests as skeletal dysplasia in humans with achondrogenesis type 1A. GMAP-210 tethers ER derived vesicles containing newly synthesized extracellular matrix proteins en route to the Golgi apparatus. It also tethers intra-Golgi vesicles responsible for recycling Golgi enzymes that are important for modifying secretory cargo, including extracellular matrix proteins, which is required for their maturation. Following transit through the Golgi the fully modified matrix proteins are secreted and assembled into the extracellular matrix. In addition, GMAP-210 anchors IFT20 to the Golgi, which is required for the IFT20-dependent transport of cargo proteins to the cilium. This is necessary for ciliary signaling, which helps maintain the differentiation status of chondrocytes and their ability to synthesize and secrete large amount of matrix proteins. Upon loss of GMAP-210, as occurs in achondrogenesis type 1A (ACG1A), the loss of vesicle tethering at the *cis*-Golgi results in a deficit in both transport and modification of extracellular matrix proteins, which in turn causes a defective cell matrix to form outside the cells. This manifests as the severe skeletal dysplasia achondrogenesis Type 1A. There is also a loss of IFT20-dependent transport of cargo proteins to the cilium, resulting in defective ciliary function and impaired cell differentiation, such that matrix production is also decreased, further compounding the phenotype.

Mutations in GMAP-210 that result in loss of protein expression cause the severe neonatal lethal autosomal recessive disorder achondrogenesis type 1A (ACG1A) (Smits et al., 2010). ACG1A is manifest as skeletal dysplasia, with shortening of the bones and reduced ossification, accompanied by craniofacial abnormalities and other skeletal defects, which is similar to the phenotype seen in knockout mice (Molz and Spycher, 1980; Smits et al., 2010). Within ACG1A chondrocytes, there is swelling of the ER and Golgi fragmentation, accompanied by reduced secretion of extracellular matrix (Smits et al., 2010; Wehrle et al., 2018, 2019) (Figure 4). This is further compounded by reduced differentiation of the chondrocytes, resulting in reduced cell numbers, such that matrix protein secretion is greatly reduced in the bone growth plates (Wehrle et al., 2019). The results are consistent with human GMAP-210 playing an important role in the modification and trafficking of extracellular matrix proteins, as seen in the mouse. It is also likely that ciliary impairment might contribute to the ACG1A phenotype, possibly via control of chondrocyte differentiation, although this remains to be confirmed. Recently, a second genetic cause of ACG1A has been identified, with mutations in the lamin B receptor (LBR) shown to cause an identical clinical phenotype to that seen upon loss of GMAP-210 (Wehrle et al., 2018). LBR is localized to the ER and nuclear envelope, where it not only binds to lamin B, but is also a key enzyme in the production of cholesterol (Tsai et al., 2016). The loss of LBR results in reduced synthesis of cholesterol (Tsai et al., 2016), which is known to be important for secretory protein traffic (Stuven et al., 2003; Ridsdale et al., 2006). Hence, depletion of cholesterol upon LBR deficiency impairs transport within the secretory pathway, pointing to a common pathogenic mechanism to that seen upon loss of GMAP-210 (Wehrle et al., 2018). Mutations in LBR are also associated with a different skeletal disorder known as Greenberg syndrome (Greenberg et al., 1988). The differences in phenotype between ACG1A and Greenberg syndrome may be explained by residual expression of truncated LBR in the latter, which, although it remains to be demonstrated, could induce additional cellular phenotypes to those seen upon the complete loss of LBR in ACG1A. Nevertheless, the demonstration of altered secretory trafficking in LBR-associated ACG1A suggests the same defect may also account for, or contribute to, the Greenberg dysplasia phenotype.

Hypomorphic mutations in GMAP-210 result in a milder skeletal disorder known as odontochondrodysplasia (ODCD) (Wehrle et al., 2019). ODCD manifests as skeletal abnormalities including shortening of the tubular limb bones and scoliosis, accompanied by dental abnormalities (Goldblatt et al., 1991). The milder phenotype of ODCD compared to ACG1A is due to the residual expression of GMAP-210 in the former, allowing for a less severe impairment of secretory protein traffic, and hence a less severe effect on extracellular matrix deposition during bone development (Wehrle et al., 2019). At the cellular level, the severity of Golgi disruption and trafficking deficiency correlates well with the amount of GMAP-210 lost from cells, consistent with the notion that ODCD simply reflects a less severe form of GMAP-210 deficiency.

### GM130

Mutation of GM130 in humans appears to manifest as a neuromuscular disorder, with developmental delay, progressive microcephaly, and muscular dystrophy (Shamseldin et al., 2016). The mechanisms underlying these phenotypes remain to be determined, but based upon studies in zebrafish and mouse models it is likely defects in secretory trafficking, and possibly microtubule organization, are responsible. It should be noted that only a single patient with this disorder has been identified, and although the mutation in this patient resulted in a loss of GM130 protein, the identification of additional patients will be required to establish this condition as a GM130-dependent disorder. Reduced expression of GM130 has also been implicated in tumorigenesis (Baschieri et al., 2014; Baschieri and Farhan, 2015). GM130 levels are reduced in breast cancer, and depletion of GM130 in cancer cells increases cell migration, which may be relevant for the human disease. It has been proposed increased cell migration arises from altered Cdc42 activity downstream from RasGRF, to which GM130 normally binds.

### Other Golgi Trafficking Proteins and Human Disease

Mutations in many Golgi proteins have been identified as the cause of disease in humans (Zappa et al., 2018). Of interest here are those within the COG proteins, which form the multi-subunit COG vesicle-tethering complex that acts at several Golgi cisternae to mediate recycling of Golgi-resident enzymes within the Golgi stack (Willett et al., 2013). COG interacts with several golgins, and can also interact with Rab GTPases and SNAREs, and likely acts downstream of the golgins to facilitate the transition from golgin-mediated vesicle capture to SNARE-dependent membrane fusion (Willett et al., 2013; Witkos and Lowe, 2017). Mutation of COG subunits causes defects in the glycosylation of cargo proteins within the Golgi, which is a consequence of impaired recycling of Golgi enzymes within the Golgi stack. This manifests as various tissue specific defects grouped under the umbrella of type II congenital disorders of glycosylation (CDGs) (Zeevaert et al., 2008). The nature and severity of the phenotype depends upon the COG subunit mutated, and the nature of the mutation itself. The COG CDGs indicate that impairment of intra-Golgi trafficking can manifest as disease, raising the possibility that additional CDGs may be caused by mutation of golgins mediating intra-Golgi trafficking. The *trans*-Golgi protein GORAB, although originally described as a golgin (Hennies et al., 2008), is in fact a scaffolding protein for the COPI vesicle coat, and likely participates in vesicle formation as opposed to vesicle tethering (Witkos et al., 2019). Loss of GORAB causes the skin and bone disorder gerodermia osteodysplastica, which can be attributed to defective glycosylation of extracellular matrix proteins in these tissues, and as such can also be classified as a CDG (Hennies et al., 2008; Chan et al., 2018).

## Summary and Future Directions

As can be seen from the study of golgins, it is not trivial to extrapolate findings generated in cell models to whole organism physiology. Nevertheless, in many cases, it is possible to derive explanations for the organismal phenotype based upon knowledge gained from *in vitro* cell biological studies. Hence, in most cases we see defective secretory traffic and/or cargo protein glycosylation in the affected cell types in *in vivo* models and human patients. What is less clear is why certain cell types or cargo proteins within the organism have a greater sensitivity to loss of a particular golgin compared to another. A trivial explanation is that tissue- or cell-type specific differences in golgin expression are responsible. So, for golgins with overlapping specificity, we may envisage differences in their expression to account for differences in phenotypic severity between different tissues or cell types. This may be further complicated by the presence of different golgin transcripts in different cell types, which may vary during development and also between species. For example, GMAP-210 is expressed as several transcripts in human cells as a result of alternative splicing, with some of the variants lacking regions encoding functionally important regions of the protein (Ramos-Morales et al., 2001; Wehrle et al., 2019). Splicing of GMAP-210 varies between cell types and also during chondrocyte differentiation, consistent with it being functionally important (Wehrle et al., 2019). Interestingly, there are several GM130-related transcripts that are expressed in primates, but absent from non-primate species, probably as a consequence of genomic rearrangements that occurred during primate evolution (discussed in Munro, 2011). Hence, this golgin may have evolved to have several isoforms, whose functions have yet to be investigated.

Redundancy in golgin function is likely to occur in most cells and during most developmental stages, accounting for the lack or restricted nature of most phenotypes seen upon golgin knockout. However, the presence of an overt phenotype upon loss of many of the golgins, indicates that *in vivo*, redundancy is rarely complete. To better understand the degree of redundancy between golgins in an *in vivo* context, it will be interesting to perform combinatorial knockouts of the golgins. To date this has only been performed in mice with golgin-84 and giantin, which failed to reveal any functional redundancy between these golgins (Bird et al., 2018). This type of combinatorial approach will be easier to perform in more tractable species such as *D. melanogaster* or *C. elegans*, and based upon the findings in these species, it will also be important to explore further redundancy in vertebrate models. A good starting point might be the golgins with similar amino-terminal vesicle tethering motifs (Wong et al., 2017), which would be predicted to tether vesicles carrying similar cargo proteins. However, it is also important to consider that several of the golgins participate in functions other than vesicle tethering, and hence, in certain cases, the phenotypes observed *in vivo* will also depend upon cellular defects that are unrelated to vesicle tethering.

Another factor to consider is the nature of the cargo proteins themselves, and also the rate of their production, modification, and secretion. It is interesting that the extracellular matrix is particularly sensitive to loss of two of the golgins, GMAP-210 (Smits et al., 2010; Wehrle et al., 2018, 2019) and giantin (Kikukawa et al., 1990, 1991; Lan et al., 2016; Katayama et al., 2018). Matrix proteins tend to be bulky, complex, and highly modified within the Golgi, and they are produced at very high levels during skeletal development. It is therefore reasonable to propose that their production is intrinsically more sensitive to loss of golgin function, although why loss of only GMAP-210 and giantin manifests as a matrix phenotype is still unclear. This might suggest tethering of vesicles containing matrix proteins, or the enzymes that modify them, is mediated by these golgins. Defective trafficking to the cilium is also likely to contribute to the phenotype considering that both golgins appear to function in this process (Follit et al., 2006; Bergen et al., 2017; Monis et al., 2017), and alterations in ciliary signaling are known to impact upon skeletal development (Braun and Hildebrandt, 2017). Similarly, loss of GM130 manifests as a phenotype in Purkinje neurons, even though the other *cis*-Golgi golgins are abundantly expressed in these cells (Liu et al., 2017). Again, this cell-type sensitivity may reflect the high secretory capacity of Purkinje neurons, especially during dendritic growth and maintenance, but also suggests that tethering of vesicles carrying Purkinje cell dendrite-specific cargoes is particularly reliant upon GM130. However, it could also reflect roles for GM130 in other cellular processes such as microtubule nucleation (Rivero et al., 2009), which is known to be important for dendrite morphogenesis (Kapitein and Hoogenraad, 2015). Further studies will be required to address these possibilities, and to determine the degree to which impaired tethering of vesicles carrying different cargoes can account for the *in vivo* phenotypes seen upon loss of different golgins. An attractive approach would be to recapitulate the vesicle-tethering assay that has been used so effectively *in vitro* (Wong and Munro, 2014; Shin et al., 2017; Wong et al., 2017), in an *in vivo* context, to identify the spectrum of cargoes in different tissues that are tethered by the different golgins.

Another factor to consider when it comes to studying golgins *in vivo* is the propensity for cells and organisms to adapt to the chronic loss of expression of a particular protein. This has been clearly observed in giantin knockout models, where there are compensatory changes in the expression of numerous Golgi enzymes, which is likely to ameliorate the cellular and organismal phenotype (Stevenson et al., 2017). Similarly, analysis of GMAP-210-deficient chondrocytes has revealed changes in the expression levels of both secreted extracellular matrix proteins and Golgi resident trafficking machinery, which includes other golgins (Bird et al., 2018). Hence, the manifestation of the phenotype is a consequence of not only the trafficking defect directly induced by loss of the golgin itself, but also the cell’s response to this defect. This complicates experiments to directly analyze golgin function *in vivo*, but is less of a concern when it comes to modeling human disease, which is nearly always due to chronic loss of protein function. Although adaptive or compensatory responses can complicate interpretation of phenotypes, they can also provide new insight into golgin function. For example, changes in expression of trafficking machinery, Golgi enzymes or cargo proteins can allow for a more global understanding of how golgins function within the secretory pathway (Stevenson et al., 2017; Bird et al., 2018). Other changes within the transcriptome more generally can also provide a way to better understand the cellular consequences downstream from the loss of a particular golgin, which may lead to the development of new hypotheses regarding golgin function within cells and in the organism. In terms of more directly analyzing golgin function *in vivo*, methods to acutely remove the protein, such as the auxin-inducible degron system should prove to be informative (Nishimura et al., 2009). This approach is now more tractable given the advent of CRISPR-Cas9 technology to knock-in the degron tag.

It is interesting that spermatogenesis is particularly sensitive to loss of golgin function. Three golgins have been shown to be important for acrosome formation (GM130, golgin-160, TMF) (Lerer-Goldshtein et al., 2010; Bentson et al., 2013; Han et al., 2017), underlining the importance of Golgi-dependent trafficking for the biogenesis of this organelle. It is possible that other golgins also contribute to this process. Golgin-84 is highly enriched in the testis (Bascom et al., 1999), consistent with a role in spermatogenesis, although it does not appear to be absolutely required for this process as male golgin-84 knockout mice are fertile (McGee et al., 2017). GMAP-210 has been localized to pro-acrosomal vesicles during spermatogenesis, suggesting a role in acrosome biogenesis (Kierszenbaum et al., 2011), but the functional requirement for GMAP-210 in this process has yet to be tested in an animal model. Interestingly, germline specific loss of the GMAP-210 interaction partner IFT20 causes male infertility, although whether acrosome biogenesis is affected in these mice has not been determined (Zhang et al., 2016). For remainder of the golgins, we await the development of suitable animal models to assess their involvement in spermatogenesis. For those golgins that function in spermatogenesis, it will be important to better define the mechanisms by which they act. This may help explain why some golgins appear dispensable for this process (golgin-84 and giantin) (McGee et al., 2017), whereas others are not (GM130, golgin-160, TMF) (Lerer-Goldshtein et al., 2010; Bentson et al., 2013; Han et al., 2017). It would also provide new mechanistic insight into the process of acrosome formation. In addition to differences in testis-specific expression, we may expect pro-acrosomal vesicle trafficking to have a functional requirement for certain golgins over others, and there is also the possibility of redundancy in golgin function during this process. Further investigation will be required to distinguish between these possibilities.

## Author Contributions

ML devised and wrote the manuscript.

## Conflict of Interest Statement

The author declares that the research was conducted in the absence of any commercial or financial relationships that could be construed as a potential conflict of interest.
